# Understanding the Specificity of Human Galectin-8C Domain Interactions with Its Glycan Ligands Based on Molecular Dynamics Simulations

**DOI:** 10.1371/journal.pone.0059761

**Published:** 2013-03-29

**Authors:** Sonu Kumar, Martin Frank, Reinhard Schwartz-Albiez

**Affiliations:** 1 D015, Translational Immunology, German Cancer Research Center, Im Neuenheimer Feld 280, Heidelberg, Germany; 2 Biognos AB, Gothenburg, Sweden; Faculdade de Medicina, Universidade de São Paulo, Brazil

## Abstract

Human Galectin-8 (Gal-8) is a member of the galectin family which shares an affinity for β-galactosides. The tandem-repeat Gal-8 consists of a N- and a C-terminal carbohydrate recognition domain (N- and C-CRD) joined by a linker peptide of various length. Despite their structural similarity both CRDs recognize different oligosaccharides. While the molecular requirements of the N-CRD for high binding affinity to sulfated and sialylated glycans have recently been elucidated by crystallographic studies of complexes with several oligosaccharides, the binding specificities of the C-CRD for a different set of oligosaccharides, as derived from experimental data, has only been explained in terms of the three-dimensional structure for the complex C-CRD with lactose. In this study we performed molecular dynamics (MD) simulations using the recently released crystal structure of the Gal-8C-CRD to analyse the three-dimensional conditions for its specific binding to a variety of oligosaccharides as previously defined by glycan-microarray analysis. The terminal β-galactose of disaccharides (LacNAc, lacto-N-biose and lactose) and the internal β-galactose moiety of blood group antigens A and B (BGA, BGB) as well as of longer linear oligosaccharide chains (di-LacNAc and lacto-N-neotetraose) are interacting favorably with conserved amino acids (H53, R57, N66, W73, E76). Lacto-N-neotetraose and di-LacNAc as well as BGA and BGB are well accommodated. BGA and BGB showed higher affinity than LacNAc and lactose due to generally stronger hydrogen bond interactions and water mediated hydrogen bonds with α1-2 fucose respectively. Our results derived from molecular dynamics simulations are able to explain the glycan binding specificities of the Gal-8C-CRD in comparison to those of the Gal-8N -CRD.

## Introduction

Galectin 8 (Gal-8) is a member of the evolutionary conserved family of galectins which share a high affinity for β-galactosides [1], [2], [3]. The evolutionary history of galectins can be followed up by several lines of evidence, such as galectin encoding genes, exon-intron organization and sequence comparison of carbohydrate recognition domains (CRD) [4] . Among the galectins, Gal-8 belongs to the group of tandem-repeat galectins which consist of an N- and a C-terminal carbohydrate recognition domain (N-CRD, C-CRD) joined by a linker sequence of various lengths [5], [6]. Various biological roles have been ascribed to galectins with regard to modulation of cellular behaviour ranging from proliferation, apoptosis, differentiation to migration and, in a wider context, from tissue differentiation, immunity, inflammation to tumor development [1], [7]. Of particular interest are the tandem repeat galectins having two CRDs with apparently different binding capacities for oligosaccharides. For instance, Gal-9 and Gal-8 have been described as modulators of T lymphocyte activities [8], [9]. The tandem repeat of Gal-8 induces proliferation of T lymphocytes whereas single N- or C-CRDs of Gal-8 were not able to do so [9]. Analysis of a large variety of carbohydrate sequences for their binding to Gal-8 revealed that the N- and the C-CRD of Gal-8 have different affinities for oligosaccharides. While the N-CRD has in general better binding constants than the C-CRD [10] and a preference for sialylated and sulphated oligosaccharides, the C-CRD has a preference for non-sialylated oligosaccharides like polylactosamine and the blood group A (BGA) and B (BGB) glycan structures [10], [11], [12], [13], [14]. The differential binding capacity of the two Gal-8 CRDs has inspired experiments to clarify their distinct functional roles. It was speculated that the structural prerequisite of the Gal-8 molecule to dimerise is situated in the N-CRD [12]. The C-CRD binds to cell surface residues and by that induces phosphatidyl serine exposure entailing intracellular signalling. In another study the preference of C-CRD for blood group antigens was proposed to have an impact on the immunoprotection against bacteria expressing blood group B oligosaccharides [15].

It is obvious that different architecture and also dynamics of CRDs and, in particular, the binding pockets, influence the biological properties of the galectins. Therefore several groups have studied the mechanisms of carbohydrate binding characteristics of galectins in thermodynamic models and the requirements for specific carbohydrate binding as deduced from the tertiary protein structure of galectins by computational molecular dynamics (MD) modeling [16], [17], [18], [19], [20]. It has been suggested that a decisive factor for differences in affinity is the balance between the strength of the galectin-sugar hydrogen bonds and water mediated hydrogen bonds between the galectin and the sugar [16], [21], [22]. Although the 3D structures of the galectin CRDs have a similar fold, their amino acid sequence identity is rather low [17]. These differences in amino acid properties are responsible for the different binding of glycans to the CRDs. In a recent study the crystal structure of the N-CRD of Gal-8 was solved and the precise binding mechanisms of the tertiary protein structure for specific oligosaccharides was elucidated with regard to the respective amino acids of the binding pocket involved [23]. Three-dimensional structures of the C-CRD of Gal-8 were solved without ligands by NMR (PDB ID: 2YRO) and by X-ray crystallography without (PDB ID: 3OJB and 4FQZ) and with lactose as ligand (PDB ID: 3VKL and 3VKM [24]) which recently have been deposited into the Protein Data Bank [25].

We now performed a computational analysis of various modelled complexes of the Gal-8C-CRD in order to analyse binding specificities by using the crystal structure of the C-CRD (PDB ID: 3OJB). Our analysis is able to explain the molecular basis for experimental data previously obtained [10], [26], [27] concerning the high affinity binding of lactosamines and BGA and BGB oligosaccharides to the Gal-8C-CRD and further to clarify the differential binding capacities of Gal-8N- and C-CRD.

## Results

In order to understand the three-dimensional aspects of interaction between the human Gal-8C domain and specific glycans, we first aligned amino acid sequences and superimposed available three-dimensional structures of human galectins. Then, we performed MD simulations of various complexes in explicit water, analysed in detail the molecular interactions (e.g. hydrogen bonding and water bridging) and finally estimated the differences in free energy of binding using the MMGBSA approach.

### Structural Comparison of the Gal-8-C Domain with Gal-8-N and Other Galectins

**Figure 1:**
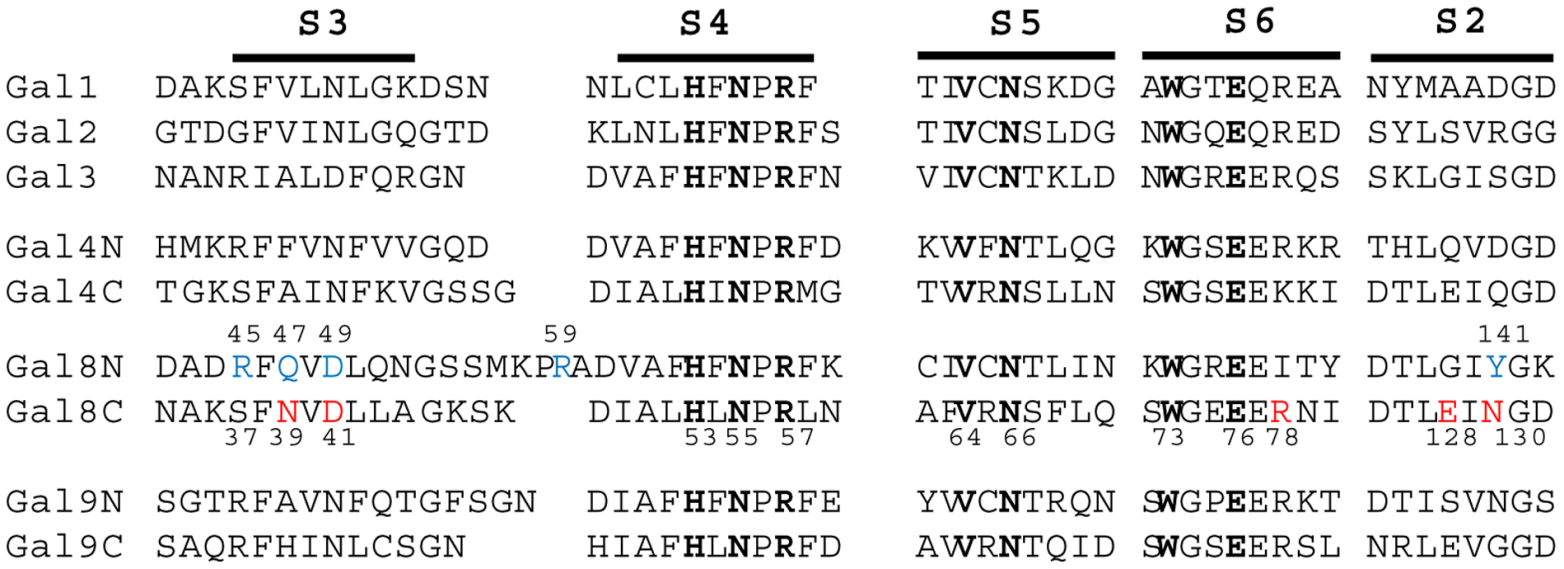
Multiple sequence alignments of the human galectin members. Conserved amino acids are shown in bold, amino acids which play important roles in interactions apart from conserved residues in Gal-8C are shown in red and in blue for Gal-8N. This multiple sequence alignment was carried out by MAFFT web server [48].

**Figure 2:**
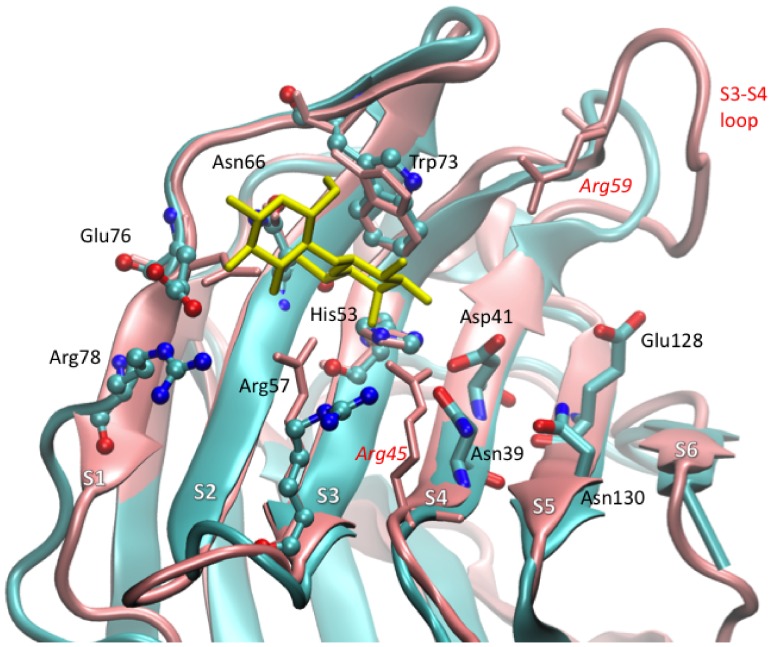
Superimposition of Gal-8N and -C domain. Ribbon representation of superimposed Gal-8N and -C domain. The N domain is shown in pink color code whereas the C domain is in cyan. Lactose is shown as stick model in yellow color. The variable loop between S3–S4 shows difference in length between Gal-8C and -N.

The multiple sequence alignments of experimentally available structures showed conservation of essential amino acids of the CRD responsible for glycan binding despite a generally low sequence identity (**Figure 1**). Interestingly, N- and C-CRD of Gal-8 share a high similarity in terms of 3-D fold (**Table 1**) which was observed by superimposing both domains using the PDBeFold web service (http://www.ebi.ac.uk/msd-srv/ssm/). Close inspection of superimposed N- and C-CRD structures revealed that a major difference is the length of the S3–S4 loop due to presence of an additional short stretch of amino acids in the N-CRD (**Figure 2**). This short stretch contains the critical arginine (R59) that makes the N-CRD domain unique for recognizing sialic acid and sulfate groups [23].

**Table 1:** Structure superimposition and degree of sequence identity.

Gal-8C	Gal-8N	Gal-9N	Gal-9C	Gal-4C	Gal-1	Gal-2	Gal-3
**RMSD (Å)**	0.96	1.15	0.75	1.21	1.53	1.46	1.12
**Sequence** **Identity**	37%	35%	41%	45%	32%	24%	38%

Three-dimensional structural alignments and sequence identity of members of the galectin family based on RMSD calculated by using the PDBeFold webserver [49].

### Interaction of Disaccharides Lactose, LacNAc and Lac-N-biose with Gal-8C

When this study was performed all available crystal structures of the Gal-8 C-CRD did not contain any ligand in the binding site. Additionally, some of the key amino acids (R57 and E76) are not in a conformation capable of establishing critical hydrogen bonds as observed in other galectin complexes, which makes the application of docking methods to generate the complexes difficult and likely to fail. Therefore we built the starting model of the lactose complex by 3D-alignment with the lactose complex of the N-CRD (PDB ID 2YXS) and transferred the ligand into the binding site of the C-CRD. The preliminary complexes for LacNAc and lacto-N-biose were built using the transferred lactose as anchor point. From here we explored different simulation conditions (see Material and Methods) in order to obtain stable trajectories for the disaccharide complexes. Finally we could sample 10 ns trajectories for all three complexes without dissociation of the ligand.

**Figure 3:**
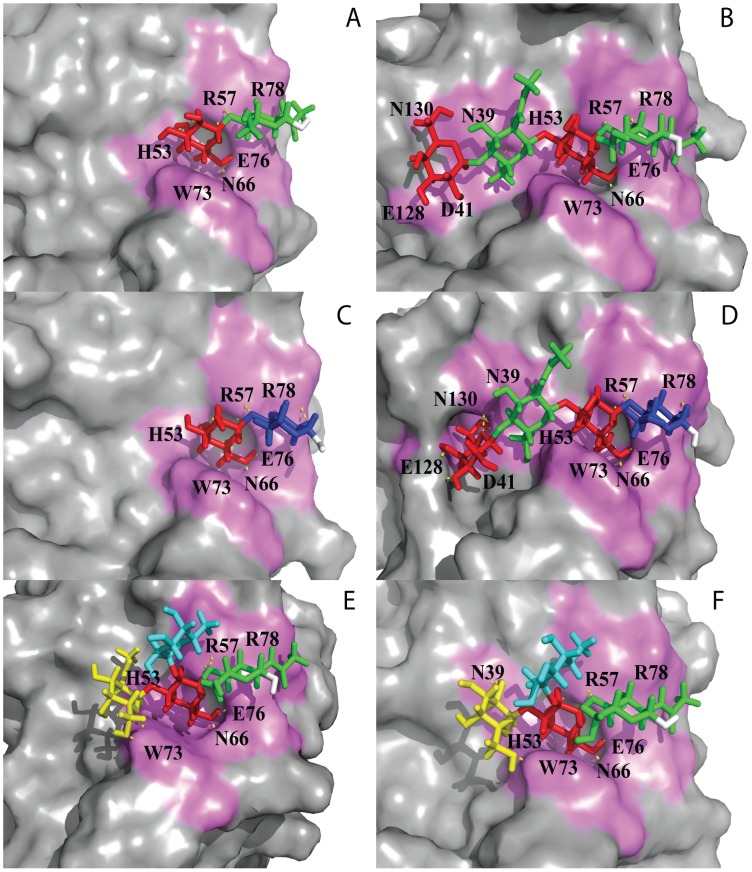
Ligand binding of the galectin-8C domain. The Gal-8C binding site with (**A**) LacNAc, II, (**B**) di-LacNAc, (**C**) Lactose, (**D**) Lacto-N-neotetrose, (**E**) BGA, and (**F**) BGB. Ligands are shown as stick models and the surface of the protein-binding site in violet color. The ligands are color-coded (β-galactose: red; N-acetyl-glucosamine: green; glucose: blue; fucose: cyan; α-galactose and α- N-acetyl-galactosamine: yellow; downstream hydroxy group: white. Hydrogen bonds are shown as yellow dotted line. A snapshot which contains a maximum number of intermolecular hydrogen bonds is displayed. See **File S1** for details of hydrogen bond interactions of each complex. The figure was designed using PyMOL Molecular Graphics System (DeLano Scientific, Palo Alto, CA).

In all three complexes the terminal β-galactose is deeply buried in the binding pocket forming hydrogen bonds with H53, R57, N66 as well as CH-π stacking of H4, H5 and H6 with the aromatic ring of W73. E76 is involved in hydrogen bonding with the monosaccharide at the reducing end (Tables S1.1, S1.2, and S1.3 in **File S1**). In case of lactose and LacNAc, O3 is hydrogen bonded to E76, whereas for lacto-N-biose it is O4. The N-acetyl group of LacNAc interacts with E78 in a similar way as found for human galectin-9C [28]. The complexes of Gal-8 C-CRD with LacNAc and lactose are shown in **Figure 3A and 3C**, respectively.

Recently, X-ray crystallography of Gal-8 C-CRD in complex with lactose was published (PDB ID: 3VKL and 3VKM [24]) which supports our MD calculations of the Gal-8C lactose complex. After superimposition of the protein backbone, the lactose ligands have a root mean square deviation (RMSD) of 1.3 Å (see **Figure S1**).

### Interaction of Carbohydrates Extended at Position 3 of Galactose (di-LacNAc and Lacto-N-neotetraose (LNnT)) with Gal-8C

In contrast to the complexes of the disaccharides, we got stable trajectories of 10 ns for all complexes shown (**Figure S2)**. For di-LacNAc (representing polyNAc) and LNnT we studied only the versions where the internal β-galactose is positioned in the primary binding site (next to W73), since these poses represent complexes in which the lactose (or LacNAc) located in the primary binding site is extended at atom O3 of galactose with LacNAc. As to be expected, the LacNAc (or lactose) in the primary binding site interacts with the same amino acids as observed in the complexes of the disaccharides. However the extended LacNAc part establishes interactions with polar amino acids N39, D41, E128, and N130 (Tables S1.4 and S1.5 in **File S1**). For comparison, an analogous LacNAc in the complex of Gal β1-4(Fucα1-3)GlcNAc β1-3Gal β1-4Glc β(LNF-III) with Gal-8N (PDB ID 3AP9) the GlcNAc residue shows also interactions with polar amino acids Q47, D49 (numbering taken from 3AP9), however the terminal Gal residue is stacking with Y141 [11]. **Figures 3B and 3D** show the Gal-8C binding pocket in complex with di-LacNAc and LNnT.

### Interaction of Blood Group Antigens with Gal-8C

BGA and BGB are branched structures due to the presence of α1-2fucose which has potential influence on the conformation of the glycosidic linkages of the neighboring residues [29]. Based on conformational energy maps derived from high-temperature MD simulations, the Fucα1-2Gal glycosidic linkage can adapt two possible low energy conformations (**Figure S3**) [30].

For further calculations we chose the global energy minima conformation (BGA: φ = 40 and ψ = 35, BGB: φ = 45 and ψ = 35). In both BGA and BGB complexes, the Gal β1-4GlcNAc moiety interacts with H53, R57, E76, R78, and N66 as in the LacNAc complex (Tables S1.6 and S1.7 in **File S1**). Binding of BGA and BGB to Gal-8C was enhanced by water mediated hydrogen bonds to the terminal sugar residue GalNAc (BGA) or Gal (BGB) and fucose (**Figures S4** **and** **S5**). In BGA the terminal GalNAc residue interacts with W73 through a hydrogen bond between O6 and Nε and the 2-acetamido group interacted through a water mediated hydrogen bond with D41 and N130, whereas in BGB the terminal Gal showed frequent hydrogen bonding to N39 and only a transient hydrogen bond between O6 and W73(Nε). The 2-, 3-, 4-OH of terminal galactose are involved in water mediated hydrogen bonds with (S37, R57), (S37, N130), and (N39, D41, N130) respectively, and additionally the ring oxygen also made a water mediated hydrogen bond with D41. The methyl group of fucose is located on top of the plane of the guanidino group of R57 which should contribute favorably to the affinity as well as various bridging waters. **Figures 3E and 3F** show Gal-8C binding pockets with BGA and BGB.

### Torsional Analysis of Bound Ligands

The average values for the glycosidic torsion angle of each protein bound ligand are shown in **Table 2**. Generally, the glycosidic linkages of the free oligosaccharides exhibit greater ranges of motion than protein bound oligosaccharides [31]. Our calculations showed that φ and ψ of the β1-4 linkage of LacNAc and lactose which interacts in the binding pocket of the Gal-8C domain remain close to the values found for complexes of galectin-3 which are 52° and 17° and 50° and 17° respectively [32]. Most of the glycosidic linkages displayed only moderate flexibility, only ψ of terminal LacNAc of lacto-N-neotetraose (LNnT) was more flexible.

**Table 2:** Torsional analysis of bound ligands.

Ligand	Linkage		Torsional angle
Lactose	β1-4	φ	53.7(8.4)
		ψ	10.4(9.0)
LacNAc	β1-4	φ	48.0(17.3)
		ψ	5.5(18.6)
Lacto-N-biose	β1-3	φ	55.8(8.3)
		ψ	14.6(9.1)
di-LacNAc	Int β1-4	φ	50.5(8.2)
		ψ	13.6(8.2)
	β1-3	φ	25.8(8.9)
		ψ	−20.2(9.5)
	Ter β1-4	φ	47.5(9.0)
		ψ	4.4(9.5)
LNnT	Int β1-4	φ	51.5(8.2)
		ψ	7.1(8.8)
	β1-3	φ	38.3(13.4)
		ψ	−23.1(13.7)
	Ter β1-4	φ	55.3(9.4)
		ψ	54.8(21.0)
BGA	β1-4	φ	45.2(8.7)
		ψ	13.6(11.4)
	α1-3	φ	−59.4(8.5)
		ψ	−53.4(8.6)
	α1-2	φ	52.3(8.4)
		ψ	21.8(10.0)
BGB	β1-4	φ	49.8(8.8)
		ψ	13.1(10.6)
	α1-3	φ	−51.0(13.4)
		ψ	−51.7(8.8)
	α1-2	φ	52.0(8.2)
		ψ	20.7(9.7

Average glycosidic torsion angles for bound ligands in the Gal-8C domain (standard deviation). φ and ψ values for glycosidic linkages using the NMR definition as H1-C1-O1-C_x_ and C1-O1-C_x_-H_x_ respectively.

### MM/GBSA Binding Energy Analysis Gal-8C Complexes

**Figure 4:**
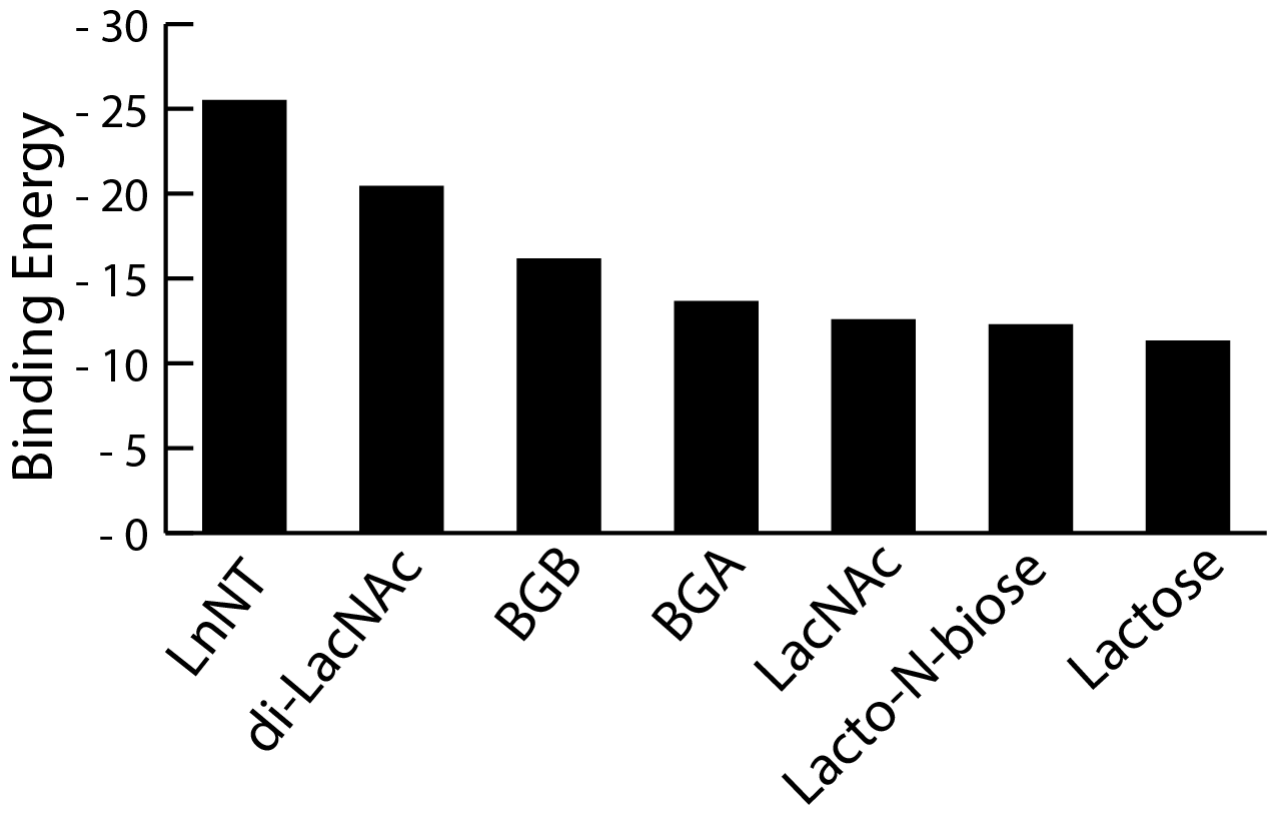
Oligosaccharides ranked by calculated binding energy towards the Gal-8C domain. The values are derived from MMGBSA energies and entropy values calculated using NMode.

Free energies of binding ΔG_binding_ are reported in **Figure 4** and details of energy contribution are shown in **Table 3** **.** **Figure 4** clearly shows lacto-N-neotetraose (LNnT) and di-LacNAc are predicted to have better interaction energies than BGA and BGB and disaccharides (LacNAc, lacto-N-biose, and lactose) on the basis of MM/GBSA binding analysis. ΔG_binding_ for all disaccharides is almost identical. Our calculations suggest that BGB has a higher affinity to the Gal-8C than BGA. Interestingly, BGA has a similar molecular mechanical interaction energy ΔE_MM_ as lactose, only because of the more favorable solvation free energy ΔG_solv_ BGA has a better ΔG_binding_ than lactose. In contrast BGB has a significantly stronger interaction energy (ΔE_MM_) and less loss of entropy (-TΔS). For the extended oligosaccharides (LNnT and di-LacNAc) our results give generally higher numbers for ΔE_MM_ and ΔG_sol_ which is mainly caused by electrostatic contributions. The more favorable electrostatic contribution in ΔE_MM_ can overcome a less favorable contribution from the polar term of solvation energy.

**Table 3:** MM/GBSA energies.

Ligand	LnNT	di-LacNAc	BGA	BGB	LacNAc	Lactose	Lacto-N-biose
ΔE_vdw_	−35.16	−35.90	−29.95	−27.35	−21.19	−16.73	−18.24
ΔE_elec_	−135.06	−115.30	−61.03	−74.36	−56.82	−74.12	−74.77
ΔE_MM_	−170.23	−151.20	−90.99	−101.71	−78.01	−90.86	93.01
ΔG_np_	−6.37	−5.76	−4.26	−4.13	−3.44	−3.17	−3.17
ΔG_pol_	121.39	106.49	57.5	68.59	50.33	63.15	65.35
ΔG_solv_	115.01	100.72	53.23	64.46	46.88	59.98	62.17
ΔG_MMGBSA_	−55.21	−50.48	−37.75	−37.24	−31.12	−30.87	−30.83
–TΔS	29.74	30.07	24.12	21.11	18.57	19.57	18.58
ΔG_binding_	−25.47	−20.41	−13.63	−16.13	−12.55	−11.3	−12.25

All values are reported in kcal/mol. ΔE_elec_, electrostatic molecular mechanical energy; ΔE_vdw_, van der Walls molecular mechanical energy; ΔE_MM_ = ΔE_elec_+ΔE_vdw_, total molecular mechanical energy; ΔG_np_, non-polar contribution to the solvation energy; ΔG_p_, polar contribution to the solvation energy; ΔG_solv_ = ΔG_np_+ΔG_p_, total solvation energy; ΔG_total_, total energy (without entropy contribution); –ΤΔS, -T (temperature)*ΔS(sum of rotational, translational and vibrational entropies); ΔG_binding_ total binding energy of the system.

## Discussion

We conducted MD simulations to obtain in-depth information about the three dimensional structural aspects for oligosaccharide binding into the fold of the Gal-8C domain. For this purpose we examined Gal-8C complexes of seven oligosaccharides which were previously found to have an affinity for the Gal-8C domain [10], [27]. Our computational analysis helps to understand experimental results with regard to the binding strength of various oligosaccharides and their specific epitopes within the oligosaccharide chain for Gal-8C.

It is evident that Gal8 displays different binding specificities in their N and C domains which in turn may influence their biological properties [12]. Alignment of galectin amino acid sequences and further superimposition of the three-dimensional structures available for several galectin CRDs including the N-domain of Gal-8 indicated that core sugar residues (H53, N55, R57, V64, N66, W73 and E76) of the recognition site are well conserved (**Figure 1**). The reason behind differences in specificity can therefore be attributed to certain critical amino acids in the vicinity of the primary binding site. The structure of the human Gal-8C domain consists of 139 residues forming a β-sandwich secondary structure consisting of six strands (S1–S6) concave and a five strand (F1–F5) convex face as shown in **Figure S6**. The concave face forms the binding pocket for carbohydrates. The entire β-sandwich secondary structure is connected through several loops and one small helix present between S2–F5 which contains important amino acids responsible for differential sugar recognition. Comparison of the S3–S4 loop between the Gal-8C and Gal-8N domains revealed that a short insertion of amino acids is present in Gal-8N which produces a longer loop than in Gal-8C, and in this loop one critical amino acid, R59, contributes to the specific recognition of sialic acid containing oligosaccharides in Gal-8N (**Figure 2**) [11]. Despite the space available for sialic acid in Gal-8C, amino acids recognizing carboxylic group of sialic acid (R59) are absent in Gal-8C as compared to Gal-8N. Amino acid R45 in Gal-8N forms a hydrogen bond with glycosidic oxygen between sialic acid and galactose which fixes the orientation of sialic acid. This Gal-8N R45 amino acid is conserved among Gal-3, Gal-9N, and Gal-9C and plays a significant role in affinity for α2-3 sialylated oligosaccharides. Instead of arginine at this position, Gal-8C has serine (S37). For Gal-8N, apart from the aforementioned conserved amino acid residues, several additional amino acids (Q47, D49, and Y141) play an important role in carbohydrate recognition [23]. In contrast, R59 is absent in Gal-8C and apart from D49 the other amino acids are absent at analogous positions and substituted by S37, N39, N130.

From our calculations, the conserved amino acids of the Gal-8C domain residing in the binding pocket interact both with type I, type II LacNAc and lactose with almost identical binding energy. Previously, similar affinities for LacNAc type II (K_d_ = 43) and lactose (K_d_ = 50) were experimentally determined [10] which is in agreement with our calculations. As usually found in galectins, also in our models of Gal-8C - carbohydrate complexes, tryptophan (W73) is involved in CH-π stacking interactions with β-galactose [33]. From previous work, the importance of arginine (R57) has been elucidated by site directed mutagenesis in that exchange of R57 to R57H in Gal8-C domain eliminated glycan recognition [12]. This is also in agreement with our observations derived from MD simulations of the disaccharide complexes. Since the crystal structure of the Gal-8 C-CRD, which was used as starting structure for the MD simulation, contains R57 in a conformation that does not allow formation of hydrogen bonds to the O3 of the glucose residue, the complexes turned out to be rather unstable until the conformation of R57 changed and the critical hydrogen bond was formed.

In summary, computational analysis of the disaccharide complexes favors the experimental results of Yoshida et al [24] regarding lactose interaction in the binding pocket of C-CRD. The presence of different glycosidic linkages (β1-3/4) in LacNAc type I and II do not seem to affect their binding with Gal-8C. The Gal-9C LacNAc complex (PDB ID: 3NV2) has similar interactions like the Gal-8C LacNAc complex with galactose (e.g. Gal O6, O4 and O5 with N248, H235, and R239 respectively) and three hydroxyl of N-acetylglucosamine with R239 and E258. This result supports previous work on galectins regarding critical interactions of Gal(O4)-H53, Gal(O6)-N66 and GlcNAc(O3)-E68 [26]. It is evident that an oligosaccharide in which a sugar residue is added at critical hydroxyl faces (e.g. Gal O4 and O6) will impede binding. The α2-6 linkage of sialic acid residue to LacNAc blocked the β-galactose and its size also causes steric hindrance within the binding pocket of both Gal-8 N- and C- domain [34]. Amino acids responsible for strong binding of α2-3 sialylated oligosaccharides are absent in the Gal-8C domain. In contrast to the Gal-8N domain which has high affinity towards α2-3sialylated lactose, due to the presence of the critical amino acid R59 [11], a stretch in the amino acid sequence in Gal-8C domain is absent at analogous position in the Gal-8N domain.

**Figure 5:**
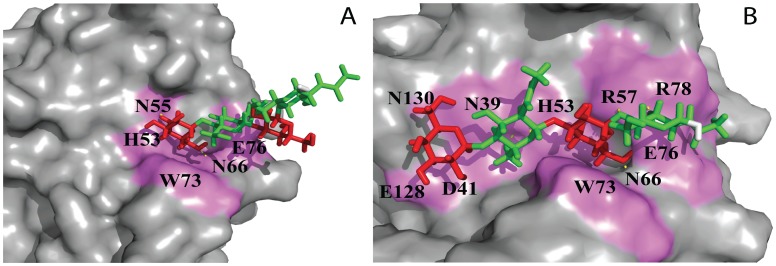
Surface representation of Gal-8C domain complexed with di-LacNAc. Carbohydrates are shown in stick. The ligands are color-coded (β-galactose: red; N-acetyl-glucosamine: green; and downstream hydroxy group: white. (**A**) Interaction of terminal β-galactose of di-LacNAc. (**B**) Interaction of internal β-galactose di-LacNAc.

The extended oligosaccharides lacto-N-neotetraose and di-LacNAc with internal and terminal β-galactose residues theoretically have two possibilities for β-galactose to interact within the core binding region of Gal8-C domain as shown in **Figure 5A and B**. As demonstrated in **Figure 5A**, binding of terminal β-galactose of the extended oligosaccharides in the primary binding site would leave the remaining sugar residues outside the protein binding pocket and hence its binding would resemble that of the disaccharide LacNAc whereas binding of internal β-galactose permits the remaining sugar residues to interact with additional amino acids (**Figure 5B**). In glycan array experiments polyLacNAc had lower binding efficiency than BGA and BGB [27] whereas in our calculations di-LacNAc was a stronger binder. It may be that the dense packing of glycans on a microarray chip causes a sterical hindrance for recognition of the internal β-galactose residues and therefore results in lower binding values. Based on the significantly increased free energy of binding for the di-LacNAc and LNnT complexes in comparison to LacNAc we conclude that our computational analysis favors the experimental results of Stowell et al [12] and Carlsson et al [10] which indicate a higher binding affinity of the Gal-8C domain for the internal rather than the terminal β-galactose moiety. By treating live cells with exo-β-galactosidase which degraded the terminal galactose, Gal-8C was shown to be still able to bind on the cell surface. Remarkably, in this set of experiments Gal-8N did not show any significant binding to polyLacNAc [12]. In contrast, LNF-III binds significantly stronger to Gal-8N than to Gal-8C [10]. This can be explained by the crystal structure of Gal-8N (PDB ID 3AP9) [23] where the terminal galactose residue of LNF-III is making strong hydrophobic stacking contact to Y141 [11], whereas based on our models of LNnT and di-LacNAc complexes the terminal galactose interacts only with polar amino acids E128, and N130 establishing only transient hydrogen bonds, which should result in lower affinity. However in Gal-8N, contrary to Gal-8C, the further extension of the linear polyLacNAc at the nonreducing end is hindered due to presence of an extended S3–S4 loop, which might explain the reduced binding of Gal-8N for polyLacNAc. In Gal-9N di-LacNAc complex (PDB ID:2ZHK) [35], the internal β-galactose moiety rather than the terminal one binds and has similar interactions (e.g. internal βGal 4O with N63, O6 with N75 and E85, and 5O with R65) which supports our Gal-8C di-LacNAc calculations.

BGA and BGB have been shown to display higher binding to the Gal8-C domain than disaccharides due to their terminal GalNAc and Gal residues respectively. Our analysis is in agreement with the experimental results of Walser et al [36] with regard to interactions of the C6 hydroxyl of terminal GalNAc in BGA with W73. The water mediated hydrogen bonds - for example involving the acetamido group of terminal GalNAc and the ring oxygen of α1-2 linked fucose - contribute to stronger binding. For BGB the OH2 group of the terminal galactose enables a strong hydrogen bond with N39 and the other hydroxyl groups of the terminal galactose are involved in various water mediated hydrogen bonds. The α1-2 linked fucose is also involved in various water mediated hydrogen bonds, but the methyl group at position 6 can also interact directly in a fovourable manner with the guanidino group of R57. In general, the α1-2 linkage of fucose in BGA and BGB antigens causes some rigidity to the structure of oligosaccharide in the binding pocket which in turn results in less loss of entropy upon binding.

Gal-8C and Gal-4C have strong affinity for BGA and BGB [15]. This is due to the presence of S37, N39 in Gal-8C and S220, A222 in Gal-4C. In particular N39 and A222 form hydrogen bond with the 2-acetamido group of BGA GalNAc. In contrast, Gal-3 [15] and Gal-9C [14] have R144, A146 and R221, H223 respectively which help in recognizing BGB more than BGA because R144 and R221 cause hindrance for 2-acetomido group of BGA GalNAc. Gal-4N, Gal-8N, and Gal-9N have R45 F47, R45 Q47, R44 A46 respectively which cause steric hindrance for BGA but not for BGB.

In conclusion, our *in silico* studies are in general agreement with the experimental data with regard to the glycan binding properties of the Gal-8C-CRD and provide valuable information about the detailed three-dimensional conditions for specific interactions with a set of non-sialylated β-glycan oligosaccharides. The MD simulations also contribute to the understanding of different binding specificities of N- and C-CRDs in tandem-repeat galectins.

## Materials and Methods

### Starting Structure

The apo structures of the human Gal-8C domain (PDB ID: 3OJB) and Gal-8N domain (PDB ID: 2YV8) were retrieved from the Protein Data Bank [25]. The amino acid numbering of Gal-8C (PDB ID: 3OJB) has been used in this study. For sequence alignments and structural superimposition with Gal-8C domain, Gal-1 (PDB ID: 1GZW) [37], Gal-2 (PDB ID: 1HLC) [38], Gal-3 (PDB ID: 1A3K) [32], Gal-4C (1X50), Gal-9N (PDB ID: 2ZHM) [35] and Gal-9C (3NV1) [28] were also retrieved.

### Preparation of Starting Protein-ligand Complexes

The saccharides used in the MD simulations for protein-carbohydrate interactions were chosen based on the carbohydrate microarray experiments previously published [10], [12], [27] and as deposited in the respective data banks of the Consortium of Functional Glycomics (CFG) [39] and affinity database [40]. The following oligosaccharides were included as ligands in our MD simulations: di-LacNAc, Lacto-N-neotetraose (LNnT), lactose, LacNAc type II (LacNAc), LacNAc type I (Lacto-N-biose), and blood group A and B oligosaccharides (BGA and BGB). (summarised in **Table 4** **)**. The ligand structures were prepared using the tleap module of AMBER tool 1.5, or the Glycam Builder server [41], the conformations of the BGA and BGB were adjusted using linkage torsion values of the global energy minimum as derived from conformational maps [30] with subsequent optimization with the molecular mechanics force field MM3 at RMS gradient of 0.001 kcal/mole/Å using the TINKER program [42].

**Table 4:** Set of oligosaccharide ligands.

Trivial Name	Nomenclature
Lactose	Galβ1-4Glcβ
LacNAc (type II LacNAc)	Galβ1-4GlcNAcβ
Lacto-N-biose (type I LacNAc)	Galβ1-3GlcNAcβ
di-LacNAc	Galβ1-4GlcNAcβ1-3Galβ1-4GlcNAcβ
Lacto-N-neotetraose (LNnT)	Galβ1-4GlcNAcβ1-3Galβ1-4Glcβ
Blood group antigen A (BGA)	GalNAcα1-3(Fucα1-2)Galβ1-4GlcNAcβ
Blood group antigen B (BGB)	Galα1-3(Fucα1-2)Galβ1-4GlcNAcβ

List of oligosaccharides used in MD simulations for study of interactions with the Gal-8C domain.

At the moment of writing all currently available crystal structures of the Gal-8 C-CRD did not contain any ligand in the binding site. Additionally some of the key amino acids (R57 and E76) are not in a conformation capable of establishing critical hydrogen bonds as observed in other galectin complexes, which makes the application of docking methods to generate the complexes difficult and likely to fail. Therefore we built the starting model of the lactose complex by 3D-alignment with the lactose complex of the N-CRD (PDB ID 2YXS) and transferred the ligand into the binding site of the C-CRD. The preliminary complexes for all other carbohydrates were built by superimposing the β-galactose residue of each ligand with the β-galactose residue of the modelled Gal-8-C lactose complex. All histidine residues (HIS) were assumed to be neutral and were protonated at the Nδ-position, hence it changed into HID. Each initial protein-ligand complex was processed for MD simulations using the tleap module of the AMBER package [43]. In this process hydrogen atoms were added to the protein, the electrostatic neutralization of the complex, and the solvation of the systems was done.

### Molecular Dynamics Simulations

MD simulations were performed for all the Gal-8C ligand bound complexes and also Gal-8C alone without any ligand in explicit solvent for 10 ns. For the simulations, the AMBER force field ff99SB was used for the protein [44], while for carbohydrates parameters were taken from the GLYCAM06 force field [45]. The complexes were solvated in a box of TIP3P water with approximate dimensions 65 Å×71 Å×63 Å using periodic boundary conditions. Firstly, energy minimization was carried out for removal of initial unfavorable contacts made by the solvent using 1000 minimization cycles (500 steps of steepest descendent and 500 steps of conjugate gradient) keeping protein backbone atoms restrained. Then, protein side chain atoms, ligands and explicit water molecules were kept unrestrained followed by unrestrained minimization with 2500 cycles (1000 steps of steepest descendent and 1500 steps of conjugate gradient) of the whole system. Secondly, the equilibration of the system was carried out by heating the system slowly from 5 to 300 K for 60 ps, followed by 100 ps of maintaining 300 K constant temperature at constant pressure of 1 atm. For the lactose complex distance restraints of <4 Å between atoms R57(CZ) and Glc(O3) as well as between atoms H53(NE2) and Gal(O4) were applied in order to stabilize the complex during the equilibration period and to force R57 to change conformation and establish a hydrogen bond to Glc(O3). Then finally, production of dynamics were performed at 300 K for 10 ns using a 2-fs time step, with the SHAKE algorithm at constant pressure of 1 atm. During the simulations, SHAKE algorithm [46] was turned on and applied to all hydrogen atoms and the particle-mesh Ewald method was used for treating the electrostatic interactions with a cutoff of 10 Å. Minimization, equilibration, and production phases were carried out by the SANDER module of AMBER 8 [43].

### Binding Energy

The relative free binding energy of Gal-8C ligand trajectories was evaluated using the Molecular Mechanics – Generalized Born Surface Area (MM-GBSA) module of AMBER 8. By using the MD trajectories collected from explicitly solvated simulations of the ligand–protein complexes, the binding free energy was computed directly from the energies of the protein, ligand and its complex components.
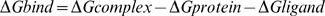



The free energies of the components were computed by separating the energies into molecular mechanical (electrostatic and van der Waals), and solvation.
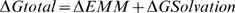


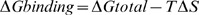



The RMSDs for the trajectory of all ligand-bound complexes were calculated using the initial minimized structure of MD production as reference. Thereafter, results (**Figure S2**) show that the RMSD of the protein has achieved a stationary phase and is always less than 2.5Å for the entire simulation length. Snapshots were extracted from the 10ns trajectories which show a distance of about 3Å between HIS53(NE2) and βGal(O4) and were analyzed using the MMPBSA.py script for enthalpy and normal modes for entropy calculations. The resulting enthalpy (ΔG_total_) and entropic (TΔS) terms were combined to give estimates of the binding free energies.

### Trajectory Analysis

The analysis of MD simulations was performed using the Conformational Analysis Tools (CAT) software (www.md-simulations.de/CAT) along with the ptraj module of AMBER tools 1.5 which was used for the superimposition of the trajectory frames and strip water from trajectory to visualize the whole trajectory with VMD. The CAT software was used to analyse each frame of the MD production runs for RMSD, hydrogen bond analysis, torsional analysis and analysis of water mediated hydrogen bonds.

All molecular graphics were done using either the PyMOL Molecular Graphics System (DeLano Scientific, Palo Alto, CA) or using VMD software [47].

## Supporting Information

Figure S1 **Overlay of our model of the Gal-8C CRD/lactose complex (in green) with the recently published X-ray structure.** (PDB ID: 3 VKL, in pink).
(TIF)

Figure S2 **RMSD plots of Gal-8C backbone with ligand complex trajectories, every 1 ps.**
(TIF)

Figure S3 **Conformation analyses of BGA and BGB.** Conformational space of glycosidic linkages of blood group antigens which represents φ and ψ of each conformation as generated during 10 ns MD simulations in gas phase. **A.** represents conformational space of blood group antigen A (BGA) and **B.** represents blood group antigens B (BGB). φ and ψ values for glycosidic linkages using the NMR definition as H1-C1-O1-C_x_ and C1-O1-C_x_-H_x_ respectively.
(TIF)

Figure S4 **BGA water mediated hydrogen bond analysis.** Water mediated hydrogen bond analyses of stationary snapshots of the protein-ligand complex as image plot. The analyses are shown for the binding site residues of Gal-8C and BGA oligosaccharide antigen. The blue color represents the average value of water mediated hydrogen bonds, i.e more than 0.5 population mean observed between the protein atoms of the residues and glycan atoms of the residue on the X- and Y-axis respectively and also labeled in graph (e.g Fuc_5O-ARG57NE; fifth oxygen of fucose interacting with NE atom of arginine 57 via water mediated hydrogen bond).
(TIF)

Figure S5 **BGB water mediated hydrogen bond analysis.** Water mediated hydrogen bond analyses of stationary snapshots of the protein-ligand complex as image plot. The analyses are shown for the binding site residues of Gal-8C and BGB oligosaccharide antigen. The blue color represents the average value of water mediated hydrogen bonds, i.e more than 0.5 population mean observed between the protein atoms of the residues and glycan atoms of the residue on the X- and Y-axis respectively and also labeled in graph (e.g Fuc_5O-ASN55OD1; fifth oxygen of fucose interacting with OD1 atom of asparagine 55 via water mediated hydrogen bond).
(TIF)

Figure S6 **The ribbon representation of human Gal-8C domain with lactose.** The concave face (S1–S6) that makes the carbohydrate recognition face and convex face consist F1–F5; both the faces are connected with several loops. Lactose is shown as stick model.
(TIF)

File S1 **Hydrogen bond analysis.** File contains Tables S1.1–S1.7. The results from hydrogen bond analyses of stationary snapshots of the protein-ligand complexes considered in the present study are summarized as image plots. Hydrogen bonds were calculated based on a geometric criterion (donor (D)-acceptor (A) distance <3.5 Å, D-H-A angle >120°). The table represents the population of hydrogen bonds observed between the atoms of the residues. The representation of amino acids and ligand in table are in three letter code and glycam nomenclature respectively. The analyses are shown for the binding site residues and ligands of the protein-ligand complexes of the Gal-8C domain with **(1)** LacNAc II, **(2)** Lacto-N-biose, **(3)** Lactose, **(4)** di-LacNAc, **(5)** Lacto-N-neotetraose, **(6)** BGA, **(7)** BGB, respectively.
(DOC)
