# Supplementary figures and images for: Understanding the Specificity of Human Galectin-8C Domain Interactions with Its Glycan Ligands Based on Molecular Dynamics Simulations
Source: PLoS One. 2013 Mar 29;8(3):e59761. doi: 10.1371/journal.pone.0059761 (PMC3612102; doi:10.1371/journal.pone.0059761)

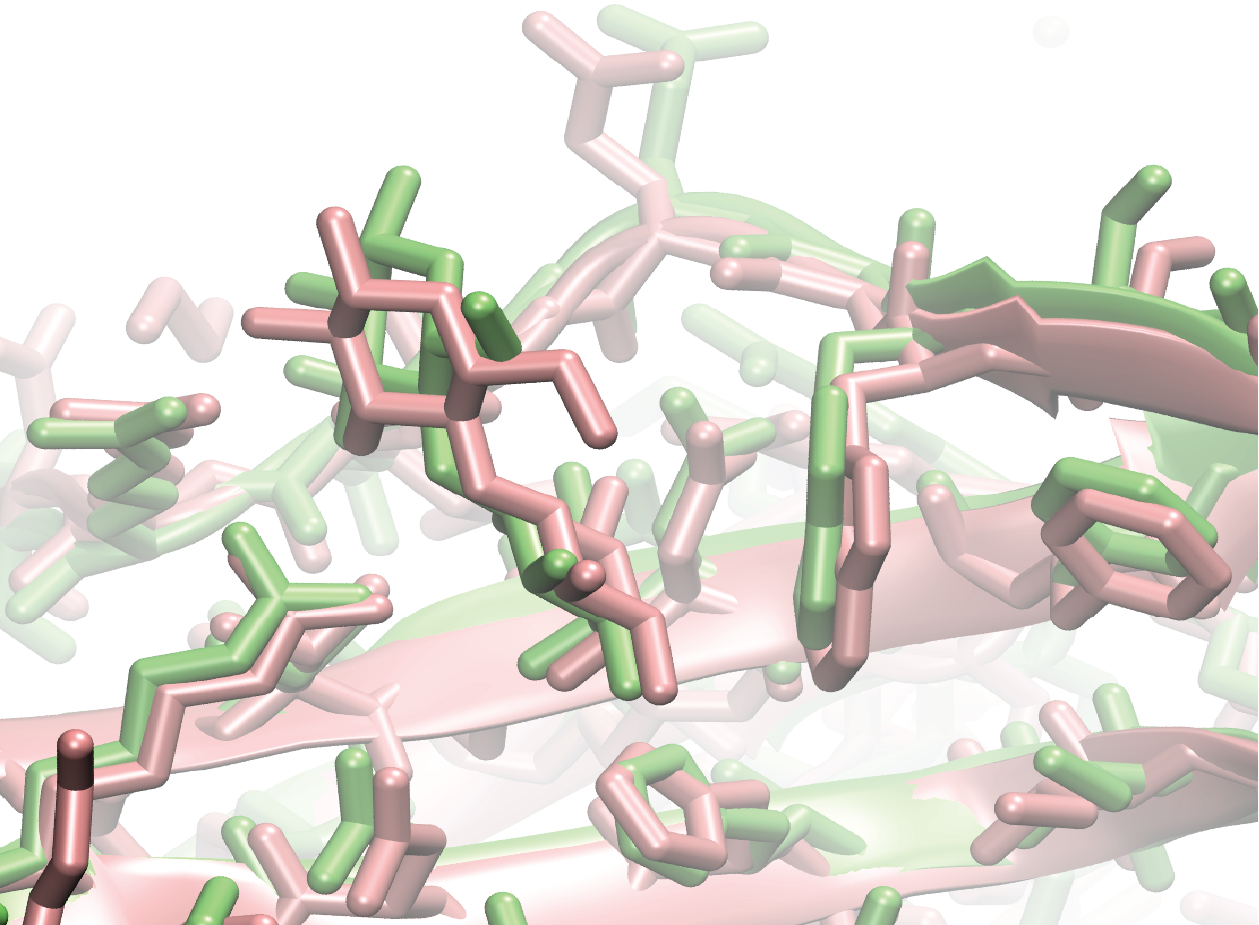

Supplement: Figure S1 — Overlay of our model of the Gal-8C CRD/lactose complex (in green) with the recently published X-ray structure. (PDB ID: 3 VKL, in pink). (TIF) [file pone.0059761.s001.tif]

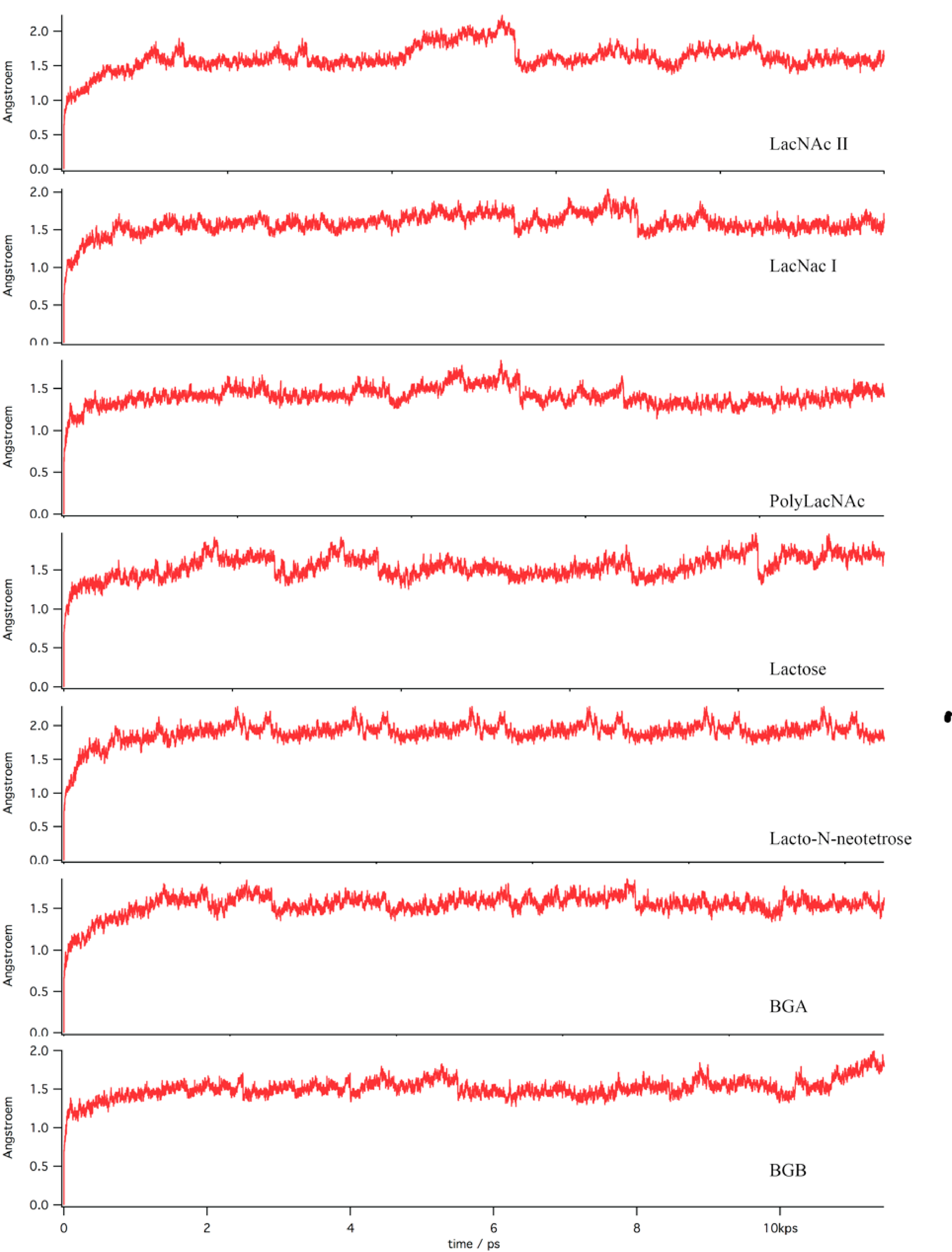

Supplement: Figure S2 — RMSD plots of Gal-8C backbone with ligand complex trajectories, every 1 ps. (TIF) [file pone.0059761.s002.tif]

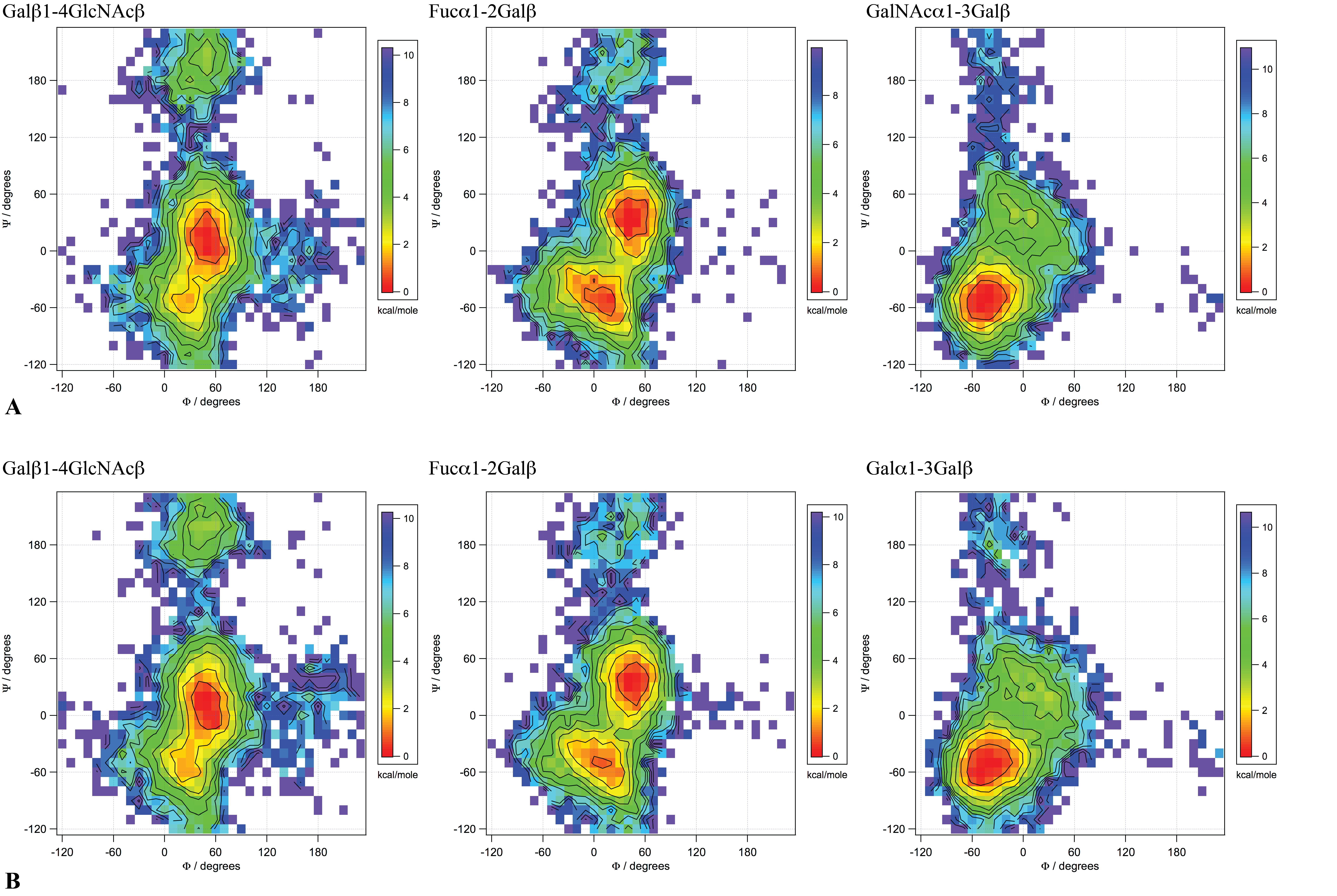

Supplement: Figure S3 — Conformation analyses of BGA and BGB. Conformational space of glycosidic linkages of blood group antigens which represents φ and ψ of each conformation as generated during 10 ns MD simulations in gas phase. A. represents conformational space of blood group antigen A (BGA) and B. represents blood group antigens B (BGB). φ and ψ values for glycosidic linkages using the NMR definition as H1-C1-O1-Cx and C1-O1-Cx-Hx respectively. (TIF) [file pone.0059761.s003.tif]

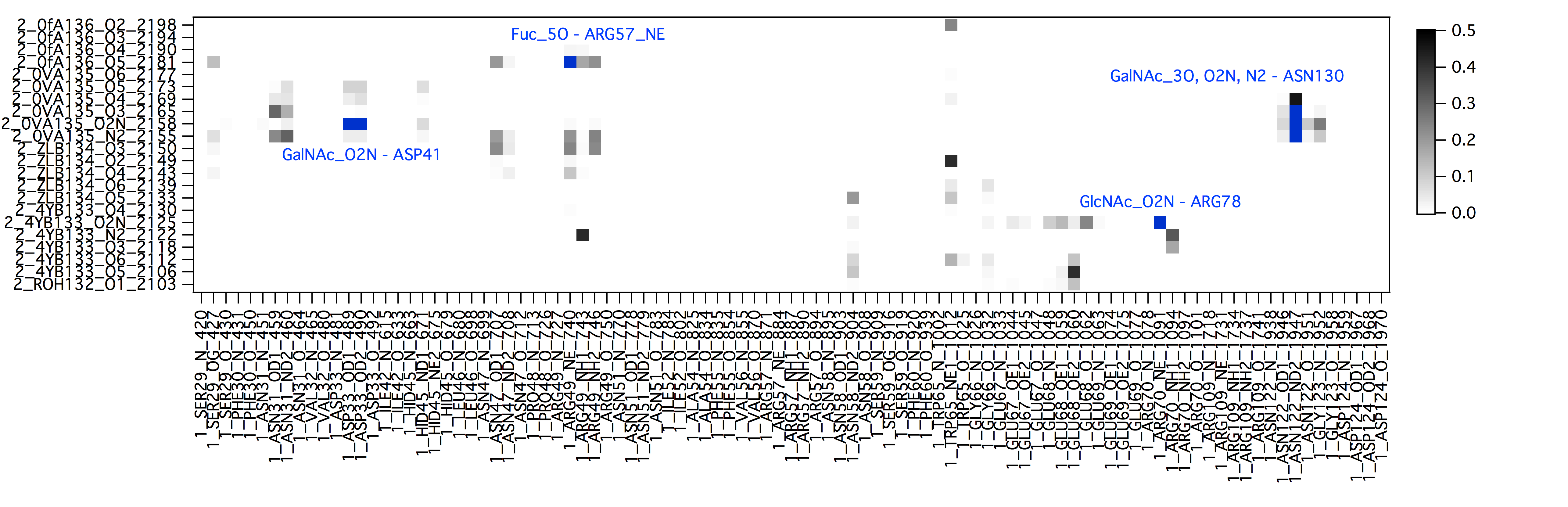

Supplement: Figure S4 — BGA water mediated hydrogen bond analysis. Water mediated hydrogen bond analyses of stationary snapshots of the protein-ligand complex as image plot. The analyses are shown for the binding site residues of Gal-8C and BGA oligosaccharide antigen. The blue color represents the average value of water mediated hydrogen bonds, i.e more than 0.5 population mean observed between the protein atoms of the residues and glycan atoms of the residue on the X- and Y-axis respectively and also labeled in graph (e.g Fuc_5O-ARG57NE; fifth oxygen of fucose interacting with NE atom of arginine 57 via water mediated hydrogen bond). (TIF) [file pone.0059761.s004.tif]

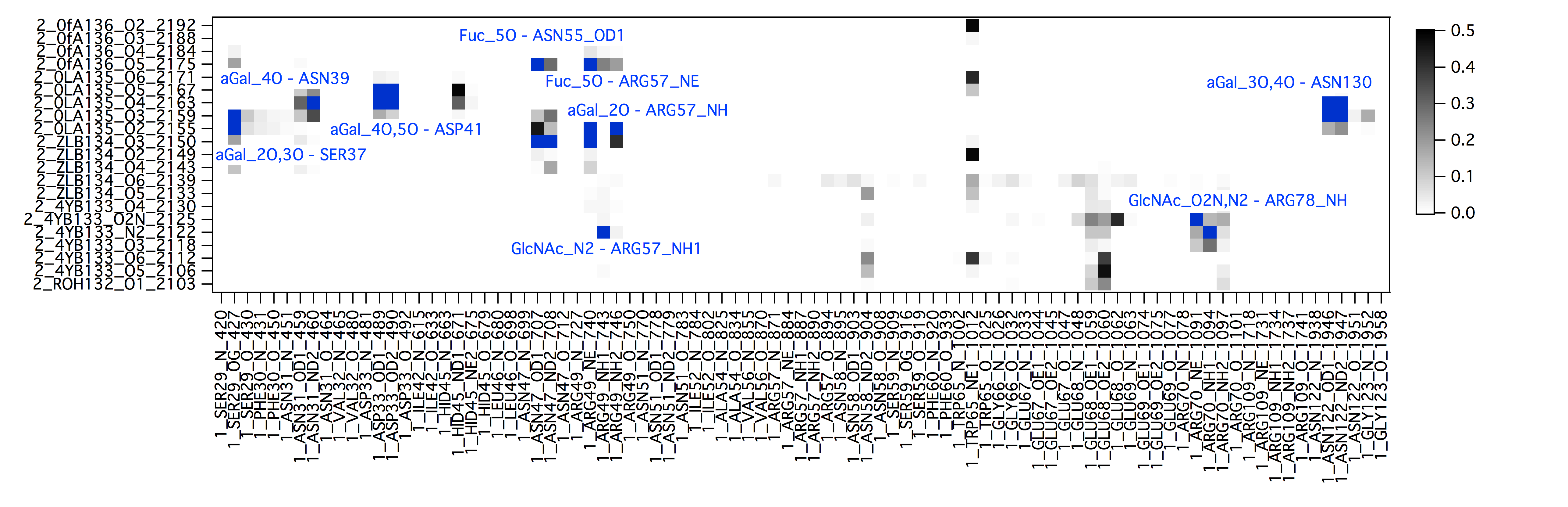

Supplement: Figure S5 — BGB water mediated hydrogen bond analysis. Water mediated hydrogen bond analyses of stationary snapshots of the protein-ligand complex as image plot. The analyses are shown for the binding site residues of Gal-8C and BGB oligosaccharide antigen. The blue color represents the average value of water mediated hydrogen bonds, i.e more than 0.5 population mean observed between the protein atoms of the residues and glycan atoms of the residue on the X- and Y-axis respectively and also labeled in graph (e.g Fuc_5O-ASN55OD1; fifth oxygen of fucose interacting with OD1 atom of asparagine 55 via water mediated hydrogen bond). (TIF) [file pone.0059761.s005.tif]

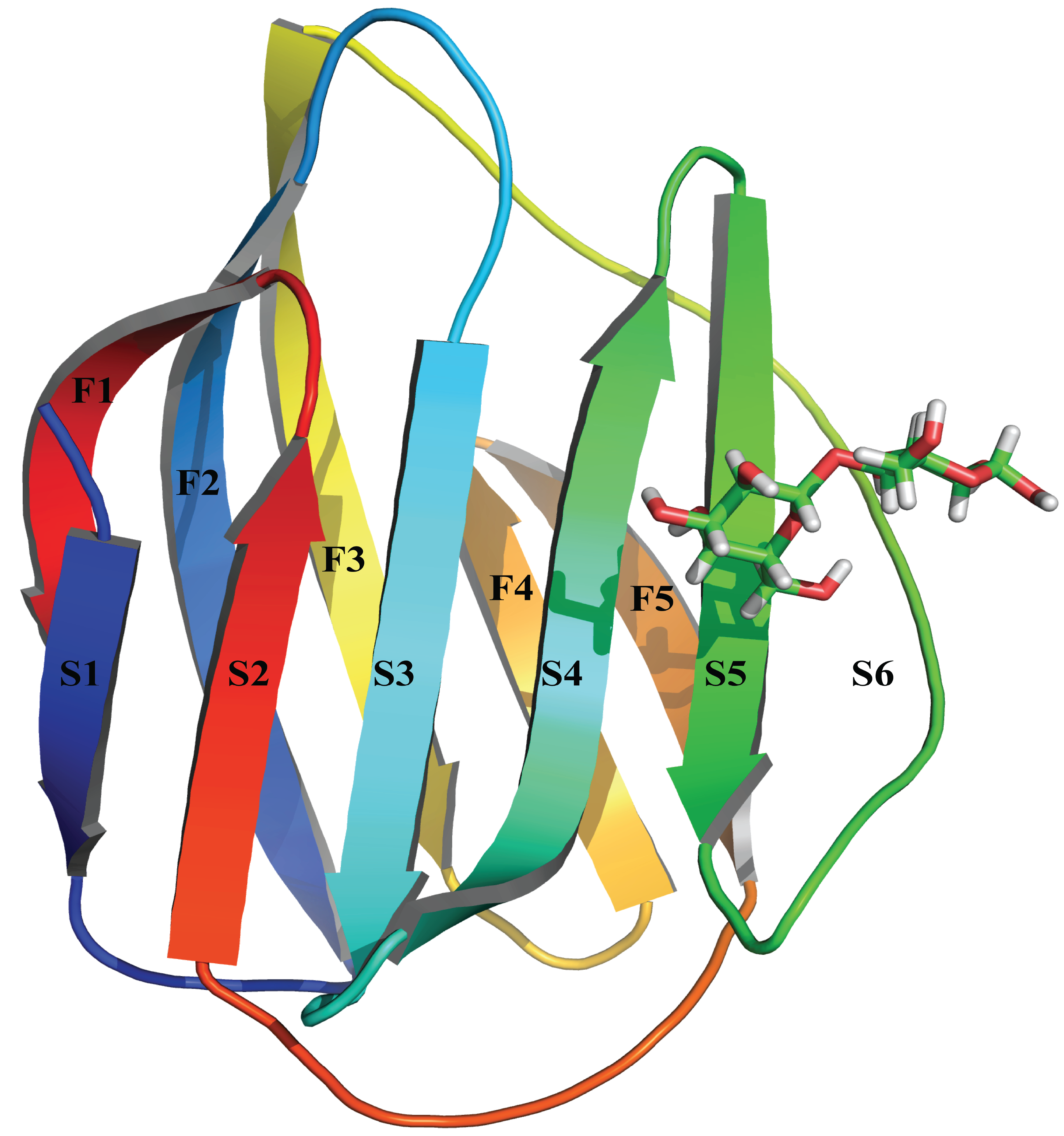

Supplement: Figure S6 — The ribbon representation of human Gal-8C domain with lactose. The concave face (S1–S6) that makes the carbohydrate recognition face and convex face consist F1–F5; both the faces are connected with several loops. Lactose is shown as stick model. (TIF) [file pone.0059761.s006.tif]
