# Supplementary material for: Understanding the Specificity of Human Galectin-8C Domain Interactions with Its Glycan Ligands Based on Molecular Dynamics Simulations
Source: PLoS One. 2013 Mar 29;8(3):e59761. doi: 10.1371/journal.pone.0059761 (PMC3612102; doi:10.1371/journal.pone.0059761)
Supplement: File S1 — Hydrogen bond analysis. File contains Tables S1.1–S1.7. The results from hydrogen bond analyses of stationary snapshots of the protein-ligand complexes considered in the present study are summarized as image plots. Hydrogen bonds were calculated based on a geometric criterion (donor (D)-acceptor (A) distance <3.5 Å, D-H-A angle >120°). The table represents the population of hydrogen bonds observed between the atoms of the residues. The representation of amino acids and ligand in table are in three letter code and glycam nomenclature respectively. The analyses are shown for the binding site residues and ligands of the protein-ligand complexes of the Gal-8C domain with (1) LacNAc II, (2) Lacto-N-biose, (3) Lactose, (4) di-LacNAc, (5) Lacto-N-neotetraose, (6) BGA, (7) BGB, respectively. (DOC) [file pone.0059761.s007.doc]

**Supporting Information**

**Understanding the Specificity of Human Galectin-8C Domain Interactions with its Glycan Ligands Based on Molecular Dynamics Simulations**

**Sonu Kumar^†^, Martin Frank^‡^ and Reinhard Schwartz-Albiez^†*^**

^†^D015, Translational Immunology, German Cancer Research Center, Im Neuenheimer Feld 280, D-69120 Heidelberg, Germany

^‡^Biognos AB, Generatorsgatan 1, 41705 Gothenburg, Sweden

KEYWORDS: Galectin-8C, MD Simulation, Glycan, Galectin Carbohydrate Interactions, Glycomics

Running Head: *glycan interactions of Gal-8C domain*

^*^Correspondence address:

Current Affiliation: German Cancer Research Center, Translational Immunology Division D015, Im Neuenheimer Feld 280, D-69120 Heidelberg, Germany, Phone: +49 (0) 6221-423713, Fax +49 (0) 6221-43737

E-mail: r.s-albiez@dkfz.de

**Table S1. Hydrogen bond analysis**

The results from hydrogen bond analyses of snapshots taken from 10 ns MD simulation of protein-ligand complexes of the Gal-8C domain with **(1)** LacNAc II, **(2)** Lacto-N-biose, **(3)** Lactose, **(4)** di-LacNAc, **(5)** Lacto-N-neotetraose, **(6)** BGA, **(7)** BGB, respectively. Hydrogen bonds were calculated based on a geometric criterion (donor (D)-acceptor (A) distance < 3.5 Å, D-H-A angle > 120°). The tables show the percentage of the trajectory the hydrogen bonds were observed. The amino acids are represented by their standard three letter code and for the cabohydrates the GLYCAM nomenclature was used.

**Table S1.1 Hydrogen bond analysis of LacNAc II pose with Gal-8C.**

| **Donor** | **Acceptor** | **Population** |
| --- | --- | --- |
| 4YB_141:O3 | GLU_76:OE2 | 99.9 |
| 0LB_142:O6 | GLU_76:OE2 | 99.7 |
| ASN_66:ND2 | 0LB_142:O6 | 82.0 |
| ARG_57:NH1 | 4YB_141:O3 | 75.3 |
| 0LB_142:O4 | HID_53:NE2 | 58.1 |
| ARG_57:NH2 | 0LB_142:O4 | 46.8 |
| ARG_57:NH2 | 4YB_141:O3 | 45.1 |
| ARG_57:NH2 | 0LB_142:O5 | 24.1 |
| 4YB_141:O3 | GLU_76:OE1 | 22.7 |
| ARG_78:NH2 | 4YB_141:O3 | 21.4 |
| 0LB_142:O4 | ASN_55:OD1 | 10.7 |
| ASN_39:ND2 | 0LB_142:O3 | 3.4 |
| ARG_57:NH2 | 4YB_141:O4 | 2.2 |
| 0LB_142:O3 | HID_53:NE2 | 1.4 |

**Table S1.2 Hydrogen bond analysis of Lacto-N-biose pose with Gal-8C.**

| **Donor** | **Acceptor** | **Population** |
| --- | --- | --- |
| 3YB_141:O4 | GLU_76:OE2 | 100.0 |
| 0LB_142:O6 | GLU_76:OE2 | 98.0 |
| ASN_66:ND2 | 0LB_142:O6 | 84.9 |
| ARG_57:NH1 | 3YB_141:O4 | 81.0 |
| 0LB_142:O4 | HID_53:NE2 | 80.7 |
| ARG_57:NH2 | 0LB_142:O4 | 40.9 |
| ARG_57:NH2 | 0LB_142:O5 | 34.9 |
| ARG_78:NH2 | 3YB_141:O4 | 30.1 |
| ARG_57:NH2 | 3YB_141:O4 | 22.5 |
| 3YB_141:O4 | GLU_76:OE1 | 16.3 |
| ASN_39:ND2 | 0LB_142:O3 | 6.8 |
| ARG_57:NH2 | 3YB_141:O3 | 2.2 |
| 0LB_142:O4 | ASN_55:OD1 | 1.3 |
| ARG_78:NH2 | 3YB_141:O6 | 1.3 |

**Table S1.3 Hydrogen bond analysis of Lactose pose with Gal-8C.**

| **Donor** | **Acceptor** | **Population** |
| --- | --- | --- |
| 4GB_141:O3 | GLU_76:OE2 | 100.0 |
| 0LB_142:O6 | GLU_76:OE2 | 99.4 |
| 0LB_142:O4 | HID_53:NE2 | 85.1 |
| ARG_57:NH1 | 4GB_141:O3 | 84.5 |
| ASN_66:ND2 | 0LB_142:O6 | 78.9 |
| ARG_57:NH2 | 4GB_141:O3 | 52.8 |
| ARG_57:NH2 | 0LB_142:O4 | 31.3 |
| 4GB_141:O3 | GLU_76:OE1 | 23.4 |
| ARG_78:NH2 | 4GB_141:O3 | 22.3 |
| ARG_57:NH2 | 0LB_142:O5 | 19.5 |
| 4GB_141:O2 | GLU_76:OE1 | 19.0 |
| ARG_78:NH2 | 4GB_141:O2 | 16.8 |
| ARG_57:NH2 | 4GB_141:O4 | 1.1 |

**Table S1.4 Hydrogen bond analysis of di-LacNAc pose with Gal-8C.**

| **Donor** | **Acceptor** | **Population** |
| --- | --- | --- |
| 4YB_141:O3 | GLU_76:OE2 | 100.0 |
| 3LB_142:O6 | GLU_76:OE2 | 99.3 |
| 4YB_143:O6 | ASP_41:OD1 | 98.6 |
| 3LB_142:O4 | HID_53:NE2 | 96.8 |
| ASN_66:ND2 | 3LB_142:O6 | 85.5 |
| ARG_57:NH1 | 4YB_141:O3 | 80.3 |
| ASN_39:ND2 | 4YB_143:O6 | 73.7 |
| ARG_57:NH2 | 3LB_142:O4 | 46.4 |
| ARG_57:NH2 | 4YB_141:O3 | 34.3 |
| ARG_57:NH2 | 3LB_142:O5 | 34.2 |
| 4YB_143:O6 | ASP_41:OD2 | 28.0 |
| ARG_78:NH2 | 4YB_141:O3 | 27.5 |
| 4YB_141:O3 | GLU_76:OE1 | 27.1 |
| ASN_130:ND2 | 4YB_143:O6 | 15.7 |
| 0LB_144:O3 | GLU_128:OE1 | 9.8 |
| 0LB_144:O3 | GLU_128:OE2 | 5.8 |
| 0LB_144:O6 | ASN_130:OD1 | 5.4 |
| 0LB_144:O2 | ASP_41:OD1 | 2.1 |
| ASN_39:ND2 | 4YB_143:O5 | 1.7 |
| ARG_57:NH2 | 4YB_141:O4 | 1.4 |
| ASN_39:ND2 | 4YB_143:O2N | 1.3 |

**Table S1.5 Hydrogen bond analysis of Lacto-N-neotetraose pose with Gal-8C.**

| **Donor** | **Acceptor** | **Population** |
| --- | --- | --- |
| 0LB_144:O6 | ASP_41:OD2 | 100.0 |
| 4GB_141:O3 | GLU_76:OE2 | 100.0 |
| 3LB_142:O4 | HID_53:NE2 | 100.0 |
| 3LB_142:O6 | GLU_76:OE2 | 100.0 |
| ASN_39:ND2 | 4YB_143:O2N | 95.0 |
| ARG_57:NH2 | 3LB_142:O4 | 95.0 |
| ARG_57:NH1 | 4GB_141:O3 | 75.0 |
| ASN_130:ND2 | 0LB_144:O6 | 65.0 |
| 0LB_144:O4 | GLU_128:OE2 | 55.0 |
| 0LB_144:O4 | GLU_128:OE1 | 50.0 |
| ASN_66:ND2 | 3LB_142:O6 | 45.0 |
| 4GB_141:O2 | GLU_76:OE1 | 35.0 |
| ARG_57:NH2 | 4GB_141:O3 | 35.0 |
| ARG_57:NH2 | 3LB_142:O5 | 35.0 |
| ASN_39:ND2 | 0LB_144:O6 | 20.0 |
| ARG_78:NH2 | 4GB_141:O3 | 15.0 |
| LYS_48:NZ | 0LB_144:O3 | 10.0 |
| ARG_57:NH2 | 4GB_141:O4 | 5.0 |
| 4GB_141:O3 | GLU_76:OE1 | 5.0 |
| ARG_78:NH2 | 4GB_141:O2 | 5.0 |

**Table S1.6 Hydrogen bond analysis of BGA pose with Gal-8C.**

| **Donor** | **Acceptor** | **Population** |
| --- | --- | --- |
| 4YB_141:O3 | GLU_76:OE2 | 100.0 |
| ZLB_142:O6 | GLU_76:OE2 | 99.4 |
| ASN_66:ND2 | ZLB_142:O6 | 99.1 |
| ARG_57:NH1 | 4YB_141:O3 | 99.1 |
| ZLB_142:O4 | HID_53:NE2 | 98.4 |
| TRP_73:NE1 | 0VA_143:O6 | 91.6 |
| ARG_57:NH2 | ZLB_142:O5 | 81.4 |
| ARG_57:NH2 | 4YB_141:O3 | 74.4 |
| ARG_57:NH2 | ZLB_142:O4 | 72.1 |
| ARG_78:NH2 | 4YB_141:O3 | 58.5 |
| 4YB_141:O3 | GLU_76:OE1 | 55.0 |
| TRP_73:NE1 | 0VA_143:O5 | 34.8 |
| ARG_57:NH2 | 4YB_141:O4 | 33.4 |

**Table S1.7 Hydrogen bond analysis of BGB pose with Gal-8C.**

| **Donor** | **Acceptor** | **Population** |
| --- | --- | --- |
| 4YB_141:O3 | GLU_76:OE2 | 99.9 |
| ZLB_142:O6 | GLU_76:OE2 | 99.4 |
| 0LA_143:O2 | ASN_39:OD1 | 91.9 |
| ASN_66:ND2 | ZLB_142:O6 | 87.1 |
| ZLB_142:O4 | HID_53:NE2 | 87.0 |
| ARG_57:NH1 | 4YB_141:O3 | 74.8 |
| ARG_57:NH2 | ZLB_142:O4 | 57.0 |
| ARG_78:NH2 | 4YB_141:O3 | 35.1 |
| ARG_57:NH2 | ZLB_142:O5 | 34.9 |
| 4YB_141:O3 | GLU_76:OE1 | 33.4 |
| ARG_57:NH2 | 4YB_141:O3 | 24.6 |
| TRP_73:NE1 | 0LA_143:O6 | 11.5 |
| ASN_39:ND2 | 0LA_143:O3 | 8.7 |
| ASN_39:ND2 | 0LA_143:O2 | 6.9 |
| ARG_57:NH2 | 4YB_141:O4 | 1.4 |
